# Correction and utilization of the washout effect in range-verification PET for particle therapy

**DOI:** 10.1007/s12194-025-01000-2

**Published:** 2026-01-05

**Authors:** Chie Toramatsu, Iwao Kanno, Taiga Yamaya

**Affiliations:** National Institutes for Quantum Science and Technology, 4‑9‑1, Anagawa, Inage‑ku, Chiba, 263‑8555 Japan

**Keywords:** Washout, Particle therapy, PET, Kinetic analysis, Tumor, Blood perfusion

## Abstract

In charged particle therapy, it is essential to verify the irradiation beam range. Thus, using positron (*β*^*+*^)-emitting nuclides which are produced in irradiated tissue, positron emission tomography (PET) has been studied, and clinically applied for in vivo range verification in particle therapy. However, a correction method for the biological washout effect is one of the fundamental issues for quantitative verification of the beam range; the irradiation-induced *β*^*+*^-emitting nuclides are affected by the pathophysiological environment such as blood perfusion. Since the biological washout effect is a tissue-specific phenomenon, extensive basic and clinical research has been conducted for modeling its kinetic process. Although considered as an undesirable factor in PET-based range validation, on the other hand, the biological washout effect may provide unique insights into the vascular status of a tumor and potentially support evaluation of the cancer pathophysiology. Consequently, this review provides a comprehensive outline of studies of the biological washout effect in particle therapy, focusing on both its correction and potential beneficial utilization.

## Introduction

In charged particle therapy, such as proton or carbon ion therapy, nuclear interactions between ion beams and the patient’s tissue result in the production of positron (*β⁺*)-emitting nuclides. In proton therapy, these nuclides arise from tissue activation, whereas in carbon ion therapy, autoactivation of the therapeutic beam itself is more dominant. The application of positron emission tomography (PET), without administration of any PET tracer, has been studied for in vivo range verification [1]. PET performed during irradiation is referred to as in-beam PET or online PET, while PET conducted after irradiation is referred to as offline PET. In either case, however, PET activity distribution does not directly reflect the delivered dose distribution due to factors such as differing physical processes and tissue elemental composition. The spatial distribution of the irradiation-induced *β*^*+*^-emitting nuclides is related to beam fluence, nuclear reaction cross sections, and target nuclide concentration distributions. The mechanism of *β⁺*-emitting nuclide production also differs depending on the species of ion beam being irradiated (e.g., protons or carbon ions): in the case of a proton beam, the mechanism involves target fragmentation alone, whereas in the case of a carbon ion beam, it involves both target and projectile fragmentation. The mechanism of production mainly affects the activity distribution of the irradiation-induced *β*^*+*^-emitting nuclides and its correlation to the deposited dose (Fig. 1). Activated target nuclei remain nearly stationary at the site of an interaction, whereas *β*^*+*^-emitting projectile fragments travel further and are accumulated at their range end which results in an activity peak. This projectile fragment activity peak is formed by the irradiation-induced *β*^*+*^-emitting nuclides from a primary therapeutic ^12^C ion beam and it is located just before the Bragg peak.

**Fig. 1:**
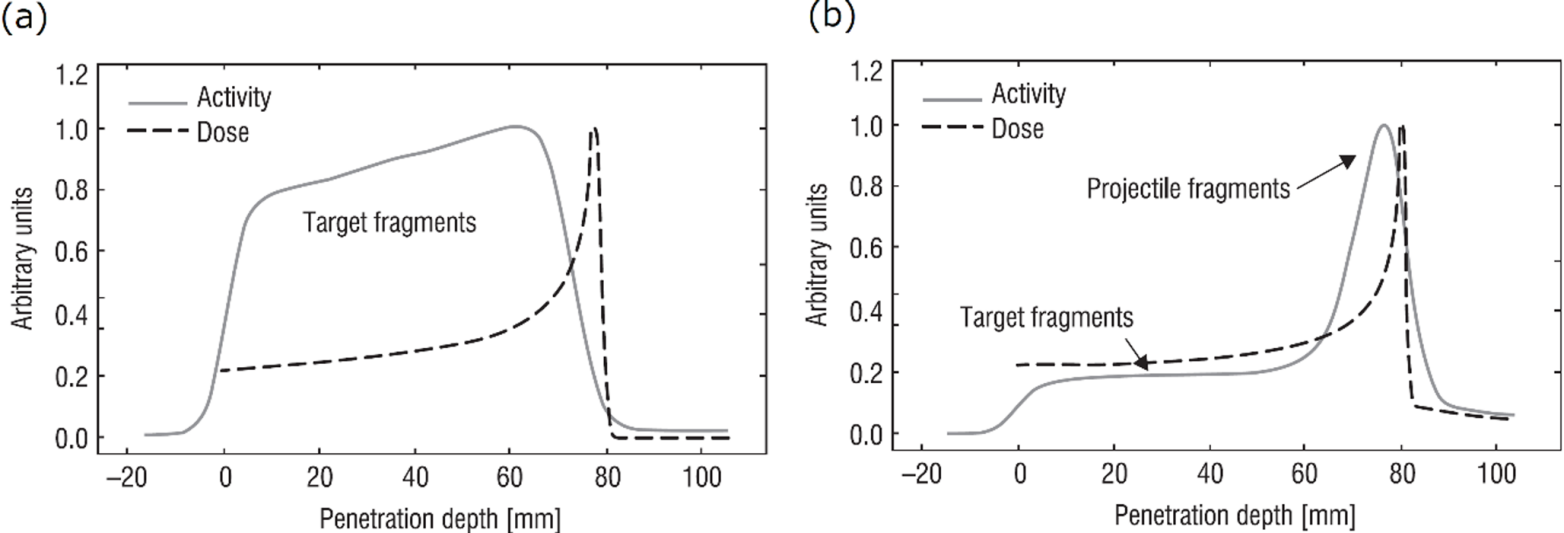
Comparison of the depth distributions of planned dose (calculation) and activity of *β*^*+*^-emitting nuclides (measurement) induced by a proton beam of 110 MeV (**a**) and a carbon ion beam of 212 MeV/u (**b**) in Lucite. Figure is reprinted from the work of Enghardt et al. [2]

Regardless of the production mechanism, dose deposition and induced activation remain in different quantities due to the different underlying electromagnetic and nuclear processes, respectively. Hence, PET-based range verification can be performed either by comparing the obtained PET activity distribution with predicted one based on the treatment plan, or by comparing the planned dose distribution with a predicted dose distribution based on the obtained PET activity distribution. For prediction of activity and/or dose distributions, methods such as Monte Carlo (MC) simulation [3] or an analytical approach [4] can be applied. These methods can achieve high precision: millimeter-level accuracy has been realized in phantom studies [5].

However, in real clinical situations, in vivo PET activity distributions change over time; *β*^*+*^-emitting nuclides in a living body eventually bind to molecules and are diffused and/or perfused by physiological processes (Fig. 2), which is referred to as the biological washout effect [6, 7]. The biological washout effect strongly degrades the correlation between dose distribution and in vivo PET activity distribution [8, 9]. Therefore, the modeling of the biological washout effect and incorporating it into a simulation or an analytical calculation is essential for PET-based range verification. The biological washout effect, however, is a dynamic and tissue-specific phenomenon, and its accurate modeling and clinical application are still under investigation.

This paper provides a comprehensive review of the biological washout effect in range-verification PET, covering data collection, kinetic modeling, clinical applications, and reported findings to date. The following sections include: descriptions of basic studies (Sect. 2); applications of basic modeling of the biological washout effect to clinical practice (Sect. 3); beneficial utilization of the biological washout effect including patient and small-animal feasibility studies (Sect. 4), and finally, the conclusions and future perspectives on in vivo range-verification PET (Sect. 5).

## Range-verification PET using RI beams

### Demonstrations in animal models

To track the *β⁺*-emitting nuclides in a living body, radioactive ion (RI) beams of *β⁺*-emitting nuclides themselves, such as ^10^C, ^11^C, and ^15^O ions, have been used instead of stable therapeutic beams, such as protons and ^12^C ions. RI beam irradiation also produces *β*^*+*^-emitting nuclides as fragments, but the activities of those fragments are almost negligible compared with that of the primary RI beam. Therefore, RI beams directly indicate the beam stopping location and enable tracking of the washout process (Fig. 2) with a signal-to-noise ratio approximately one order of magnitude higher than that of non-RI nuclide beams [10]. To evaluate the biological washout effect, it is useful to apply kinetic analysis techniques developed in nuclear medicine using RI beams irradiation. Pharmacokinetic analysis is a general technique used to quantitatively assess temporal changes in the in vivo distribution of tracers, molecules labeled with radioactive isotopes. This approach has contributed to functional diagnosis and assessment of therapeutic efficacy in cancer therapy. Molecular imaging modalities, such as diagnostic PET and magnetic resonance imaging (MRI), are employed to visualize and analyze the distribution of biological washout of tracers, thereby offering critical insights into physiological and pathological processes [11, 12].

While the biological washout effect has been viewed as an undesirable factor for PET-based range verification, it is also becoming recognized as presenting a unique opportunity to extract pathophysiological information about tumors. Clinical studies of PET-based range verification have reported noticeable changes in the biological washout rates in the irradiated areas over the course of fractionated particle therapy [13, 14]. Following these reports, small animal studies have been conducted to developing dynamic models and explore the potential utilities of the quantitative washout rate constant as a diagnostic index [15–17]. Subsequently, the feasibility of washout-based tumor diagnosis, which distinguishes hypoxic tumors from viable ones, was successfully demonstrated in tumor rat models [18, 19]. In addition, a recent study showed that the biological washout rate in tumors is dose-dependent, indicating possible factor in vascular damage due to high-dose irradiation [20]. Currently, research is on-going to explore future clinical applications of the biological washout effect, such as treatment assessment and prognosis prediction.

**Fig. 2:**
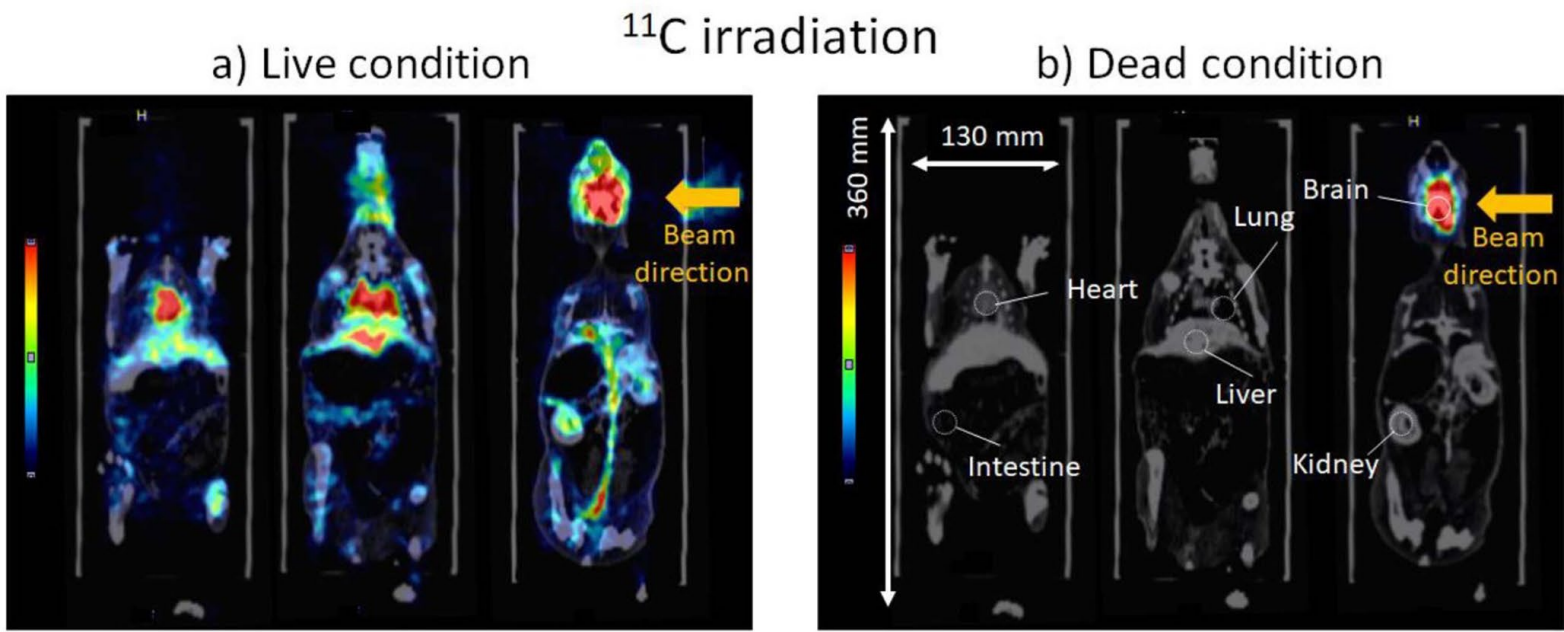
The biological washout effect, visualized by PET acquisition (10 min summation) after ^11^C ion beam irradiation to rabbit brain under the live condition (**a**) and the dead condition (**b**) (three different levels of coronal slices fused with CT images are shown). Figure is reprinted from the work of Toramatsu et al. from [21]

### Modeling the washout effect

Pioneering studies of the biological washout effect were made in the early 2000 s by Mizuno, Tomitani and their colleagues [6, 7], at the National Institutes for Quantum Science and Technology (QST) (formerly known as the National Institute of Radiological Sciences (NIRS)). ^10^C and ^11^C ion beams were irradiated on rabbit brain and thigh muscle and obtained by an in situ positron camera which consisted of a pair of scintillation cameras set on either side of the target. Then numerical expressions for the biological washout rate were derived with high statistical accuracy [7]. Obtained effective activity $\:{A}_{eff}\left(t\right)$ of *β*^*+*^-emitting nuclides in a living body depends on both physical decay, $\:{A}_{phys}\left(t\right)$, and biological decay, $\:{A}_{bio}\left(t\right)$, as follows:

1 $$\:{A_{eff}}\left( t \right) = {A_{phys}}\left( t \right) \cdot \:{A_{bio}}\left( t \right)$$

with multi exponential components,

2 $$\:{A_{phys}}\left( t \right) = p \cdot \:{e^{ - \lambda {\:_{phys\_10c}} \cdot t}} + \left( {1 - p} \right) \cdot \:{e^{ - \lambda {\:_{phys\_11c}} \cdot \:t}}\:$$

3 $${A_{bio}}\left( t \right) = {M_f} \cdot {e^{ - {\lambda _{biof}} \cdot t}} + {M_m} \cdot {e^{ - {\lambda _{biom}} \cdot t}} + {M_s} \cdot {e^{ - {\lambda _{bios}} \cdot t}}$$

4 $${M_f} + {M_m} + {M_s} = 1$$

and where $\:{\lambda\:}_{phys\_c10}$ and $\:{\lambda\:}_{phys\_c11}$ represent the respective physical decay constants of ^10^C and ^11^C. The parameter *p* represents the fraction of ^10^C contamination induced through the fragmentation reaction from ^11^C ion beam projectiles. In the case of ^10^C ion beam irradiation, *p* can be set to 1. $\:{M}_{f}$, $\:{M}_{m}$ and $\:{M}_{s}$ are the fractions of the named fast, medium and slow washout components, respectively. Their corresponding washout rate constants are: fast $\:{\lambda\:}_{biof}=\:ln2/{\tau\:}_{f}$; medium, $\:{\lambda\:}_{biom}=ln2/{\tau\:}_{m}$; and slow $\:{\lambda\:}_{bios}=ln2/{\tau\:}_{s}$, where $\:{\tau\:}_{f}$, $\:{\tau\:}_{m}$, and $\:{\tau\:}_{s}$, denote the biological half-lives (T_1/2_) of the fast, medium, and slow components, respectively. The fast component was explained as arising from the direct incidence of irradiated ions into blood vessels and their rapid washout by blood flow during the first few seconds after irradiation ended. The medium and slow components, with T_1/2_ of several minutes and several hours respectively, were observed. As a standard washout model, this model is referred to as the “multiple components model” or the “Mizuno model”, and has been implemented in simulations [22–24] and widely applied for clinical studies [8, 9].

**Fig. 3:**
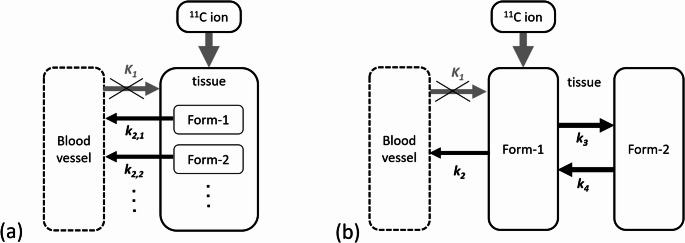
Kinetic models for irradiated ^11^C ions. **a** A single-tissue compartment with two components model. The kinetic rate constant from tissue to blood vessels for each component are denoted as *K*_*1*_, *k*_*2,1*_ and *k*_*2,2*_. **b** A two-tissue compartment model. The kinetic rate constants between compartments are denoted as *K*_*1*_, *k₂*, *k₃*, and *k₄*. In both models, (**a**) and (**b**), *K₁* that is the kinetic rate constant from blood to tissue, is omitted (due to the assumption that ^11^C ions irradiated only tissue but not blood)

The multiple components model can be considered as one of compartmental models commonly used in nuclear medicine, i.e., a single-tissue compartment model with multiple tracers. This is seen in Fig. 3 (a) which illustrates a single-tissue compartment model with two components that washout from tissue to blood vessels. Here, the component that directly incidents the blood vessels is assumed to be negligible. However, the unknown molecular forms of *β*^*+*^-emitting nuclides in vivo make it complicated to apply compartmental analysis. Hirano et al. [15] investigated applicability of compartmental model to analyze the biological washout effect of irradiation-induced *β*^*+*^-emitting nuclides. This approach allowed model parameters, such as efflux rate of *β*^*+*^-emitting nuclides from tissue to blood vessels, to be compared with those of well-known diagnostic PET tracers, including ^11^CO_2_, ^11^C-methionine, fluorodeoxyglucose (FDG), etc. They derived the biological washout rate of irradiated ¹¹C ion beam to rat brain based on the modified two-tissue compartment model (Fig. 3 (b)). Since *β*^*+*^-emitting nuclides were irradiated directly to the tissue (i.e., not administrated intravenously), the *K*_*1*_-parameter which represents the kinetic rate constant from blood vessel to tissue was omitted. The radioactive concentrations in the first ($\:{C}_{T1}$) and second ($\:{C}_{T2}$) tissue compartments, excluding *K₁*, were expressed as follows:

5 $$\:{C}_{T1}\left(t\right)=\:\frac{{I}_{1}}{{\alpha\:}_{1}-{\alpha\:}_{2}}\left\{({k}_{4}-{\alpha\:}_{1})\mathrm{exp}\left(-{\alpha\:}_{1}t\right)-({k}_{4}-{\alpha\:}_{2})\mathrm{exp}\left(-{\alpha\:}_{2}t\right)\right\}\:$$

6 $$\:{C}_{T2}\left(t\right)=\:\frac{{I}_{1}{k}_{3}}{{\alpha\:}_{1}-{\alpha\:}_{2}}\left\{\mathrm{exp}\left(-{\alpha\:}_{1}t\right)-\mathrm{exp}\left(-{\alpha\:}_{2}t\right)\right\}+{I}_{2}\mathrm{exp}\left(-{k}_{4}t\right)$$

7 $$\:{{\upalpha\:}}_{1}=\:\frac{1}{2}\left\{{k}_{2}+{k}_{3}+{k}_{4}\right\}-{\sqrt{{\left({k}_{2}+{k}_{3}+{k}_{4}\right)}^{2}-{4{k}_{2}{k}_{4}}_{\:}}}_{\:}$$

8 $$\:{{\upalpha\:}}_{2}=\:\frac{1}{2}\left\{{k}_{2}+{k}_{3}+{k}_{4}\right\}+{\sqrt{{\left({k}_{2}+{k}_{3}+{k}_{4}\right)}^{2}-{4{k}_{2}{k}_{4}}_{\:}}}_{\:}$$

Here, *I₁* and *I₂* are the initial activities of each compartment. *k₂* represents the efflux rate constant [min⁻¹] from tissue to blood, while *k₃* and *k₄* represent the influx and efflux rates between the first and second compartments, respectively (Fig. 3 (b)). Then, the total tissue radioactivity concentration was expressed as:

9 $$ \begin{aligned} C_{T} \left( t \right) & = C_{{T1}} \left( t \right) + C_{{T2}} \left( t \right) \\ & = \:\frac{{I_{1} }}{{\alpha \:_{1} - \alpha \:_{2} }}\left\{ \begin{gathered} (k_{3} + k_{4} - \alpha \:_{1} )\exp \left( { - \alpha \:_{1} t} \right) \hfill \\ - k_{3} + k_{4} - \alpha \:_{2} )\exp \left( { - \alpha \:_{2} t} \right) \hfill \\ \end{gathered} \right\} \\ & + I_{2} \exp \left( { - k_{4} t} \right) \\ \end{aligned} $$

Initially, *β⁺*-emitting nuclides in living tissue exist in a free ion form, but they may rapidly bond with nearby ions or molecules, typically within milliseconds, to form new molecules [17]. In aqueous solution, ^11^C and ^10^C ions form predominantly CO_2_, CO, and HCOOH compounds [25]. In the study by Toramatsu et al. [21], ¹¹CO₂ gas was detected in exhaled breath of rabbits following ¹¹C ion beam irradiation to the brain. This finding provided evidence that irradiated ^11^C ions react with dissolved oxygen in tissue to form ^11^C-labeled CO_2_. Other ^11^C-labeled molecules may also be formed in the tissue as a result of reactions involving irradiated ^11^C ions. The compartmental model parameters derived by Hirano et al. [15, 16] led to a low $\:{k}_{2}$ and a significantly high $\:{k}_{3}$ compared to the results of a two-tissue compartment model analysis for the ¹¹CO₂-tracer study in a dog brain done by Buxton et al. [26]. The *k*_*2*_ derived in Hirano et al. [15] was lower than that of tracers transported by simple diffusion (e.g., H_2_O), while the *k*_3_ was about 20 times higher than *k*_4_, indicating that a portion of the ^11^C was retained within the tissue. This behavior is comparable to the efflux of tracers whose permeability is regulated by transporters, such as ^11^C-methionine used in brain tumor PET [27].

In follow-up studies to Hirano et al. [15] whole-body PET scanning of rabbit was performed with ^11^C ion beam irradiation to the rabbit brain, resulting in an image resembling a blood profile of whole body [21]. It is suggested by Fig. 2 that the irradiated ^11^C ions formed ^11^CO which binds to hemoglobin (Hb) with approximately 200 times greater affinity than oxygen [28], resulting in the visualization of a blood profile-like image.

## Correction of the washout effect

Biological parameters deduced from small animal studies are not necessarily applicable to human metabolism. Applicability of biological washout models developed through animal irradiation experiments to clinical settings has been explored in clinical settings through patient studies. In patient studies, a computed tomography (CT)-based MC framework has been constructed to predict dose and/or distribution of the irradiation-induced *β⁺*-emitting nuclides [9, 29, 30]. The elemental composition of the tissue was quantified by converting the Hounsfield units (HUs) of the Digital Imaging and Communications in Medicine (DICOM) CTs using the stoichiometric calibration table of Schneider et al. [30]. Also, thresholds on the HU were set to identify each tissue type, such as fat, soft tissue, compact bone, soft bone, cortical bone, and muscle, in each patient’s CT image. Based on those segmented maps, an attempt was made to assign biological washout components to each tissue type to improve modeling accuracy. For example, a slow component was assigned for the low-perfusion area such as fat and compact bone, while a medium washout component was assigned to soft tissue [9]. Since the fractions as well as the washout rate constant are unknown in human tissues, those parameters have been assigned based on animal study results.

### Carbon (^12^C) ion irradiation

In-beam PET-based range validation was first implemented into routine clinical practice at the carbon ion treatment facility of GSI (Helmholtzzentrum für Schwerionenforschung Darmstadt, Germany) [31, 32]. In clinical data reported by Helmbrecht et al. [32], the biological washout modeling was based on the modified multiple components model [7] for application to clinical data of head and neck (H&N) treatment with ^12^C ion beams. PET scanning was performed from the end of the irradiation for about 20 min. Physical decay, $\:{A}_{phys}\left(t\right)$, and biological decay, $\:{A}_{bio}\left(t\right)$, were expressed as follows.

10 $$ A_{{phys}} \left( t \right) = \mathop \sum \limits_{i} a_{i} \cdot e^{{ - \lambda _{i} \cdot t}} + b $$

11 $$ A_{{bio}} \left( t \right) = M_{f} e^{{ - \lambda _{{biof}} \cdot t}} + B_{f} + M_{m} \cdot e^{{ - \lambda _{{biom}} \cdot t}} + B_{m} $$

Here, *i* denominates a specific nuclide, *λ*_*i*_ is its decay constant, and *a*_*i*_ is the relative abundance of the *i-*th nuclide at the end of the irradiation. The symbol *b* represents activity, which can be considered constant during the PET scan time (20 min post-irradiation). The slow component was excluded since the 20 min scan time was insufficient to detect it with the T_1/2_ of 2–3 h. Instead, the parameters $\:{B}_{f}$ and $\:{B}_{m}$, which represent fractions of the activity unaffected by the fast and medium components, were incorporated into Eq. (11). In the reported clinical data [32], no fast component (T_1/2_ of a few seconds) was observed, while the medium washout component was observed within the expected range found in animal study (T_1/2_ of a few minutes) [7]. The fast component was speculated in animal studies to be direct incidence of irradiated ions into blood vessels. In human cerebral circulation, the mean cerebral blood volume (CBV) is 3.8 ± 0.7 ml/100 ml and the mean cerebral blood flow (CBF) is 44.4 ± 6.5 ml/100 ml/min [33], the mean transit time (MTT = CBV/CBF) is 5.1 s, which agrees with the expected fast component. However, since the blood volume is only a few percent of the human brain volume [33], the probability for this event is considered low and it was negligible in the reported clinical data. Helmbrecht et al. [32] concluded that their clinical study results did not conflict with the basic principle of the multiple compartments model obtained from animal study [7].

Nischwitz et al. [34] performed an in vivo range-verification in 20 patients with glioma treated with ^12^C ion or proton. In their study, the multiple components deduced from animal and patient studies [7, 9] was considered. They concluded that, in carbon therapy, the range deviation between MC simulation and PET measurement, was within the commonly used safety margins of commonly 3–5 mm in treatment planning. In contrast, for proton therapy, the PET measurement results deviated more from the simulation predictions and further adjustments to the simulation were required. They discussed main uncertainties originating from the simple CT-based patient model and from the standard biological washout model, which considered the brain as a homogeneous tissue. Further adjustments to the simulation are required for proton irradiation and anatomical situations require particular attention to ensure accuracy as described below in Sect. 3.2 and 3.3.

### Proton irradiation

In a proton beam, the spatial correlation between the dose and PET activity range is poorer than in a ^12^C ion beam due to the lack of projectile fragments (Fig. 1). However, PET-based monitoring of proton therapy remains feasible, as the available signals are estimated to be up to twice as intense as those the ^12^C ion beam [35]. A clinical study of PET-based range verification on proton therapy of H&N tumors was conducted at Massachusetts General Hospital (MGH) [9]. In their offline PET study, the fast and medium components have already decayed at the time of imaging. The slow biological washout effect was introduced as a space- (i.e., tissue) and time-dependent weighting factor of physical activity. Values of the relative fraction $\:{M}_{s}$, and biological decay constant $\:{\lambda\:}_{bios}$ are, however, unknown for the various isotopes in human tissues. Thus, parameters based on the results of animal studies were assumed. For the sake of simplicity, tissue was classified into low (fat and compact bone), intermediate (soft bone), and normal perfusion (all remaining tissues) depends on HU value, and neglect differences between the metabolisms of different isotopes. In patients with ocular melanoma, reduced perfusion was assumed within the eye, whereas an average value was used for the surrounding tissue. As the result of applying the correction for the biological washout effect, the predicted PET activity distributions showed an average quantitative agreement of 5–30% with the measured distributions across both spatial and temporal domains [9]. Their approach, however, had several limitations, the main one being the following: the parameters were adopted from carbon ion beam studies, whose applicability to proton beam studies is questionable [36] since ^15^O ions are the dominant source of the PET signal right after proton irradiation. Therefore, they investigated how the choice of the projectile (autoactivating or non-autoactivating) influences the washout coefficients, as described in Sect. 3.3.

### Comparison of carbon ion and proton irradiation

Ammar et al. [36] reported the first experimental study investigating differences in biological washout rate coefficients dependence on projectile type. Their experiment was carried out at the Heidelberg Ion Therapy Center (HITC) to directly compare the biological washout rate coefficients of the irradiation-induced *β*^*+*^-nuclides in mice brain following irradiation with ^12^C ion and proton beams. Since offline PET scans were performed in a separate room, there was a transfer time of several minutes, and hence, the fast component was excluded from the equation of $\:{A}_{eff}\left(t\right)$. Instead, medium and slow component functions were considered. Under the assumption that the dominant signal contributions came from two isotopes ^11^C and ^15^O, $\:{A}_{eff}\left(t\right)$ was described as follows:

12 $$ A_{{eff}} \left( t \right) = A_{{0,11C}} \cdot \:M_{s} \cdot \:\left[ \begin{gathered} A_{{0,15O}} /A_{{o,11C}} \cdot \:\exp \left( { - \lambda \:_{{15O}} \cdot \,t - t^{*} } \right) \hfill \\ + \exp \left( { - \lambda \:_{{11C}} \cdot \:t - t^{*} } \right) \hfill \\ \end{gathered} \right] \cdot \left[ \begin{gathered} M_{m} /M_{s} \cdot \:\exp \left( { - \lambda \:_{{biom}} \cdot \:t} \right) \hfill \\ + \exp \left( { - \lambda \:_{{bios}} \cdot \:t} \right) \hfill \\ \end{gathered} \right] $$

where $\:{t}^{*}$ denotes the time elapsed between the end of irradiation and start of PET acquisition. $\:{A}_{\mathrm{0,11}C}$ and $\:{A}_{\mathrm{0,15}O}$ represent the activities of ^11^C and ^15^O ions, respectively, at the start of the PET acquisition ($\:{t}^{*})$. When comparing the washout constants, a clear difference was observed between the ^12^C ion and proton beam irradiation experiments. The ^12^C ion irradiation data being described best by two components fit with a combined medium and slow washout fraction ($\:{M}_{m}+{M}_{s}$) of 0.50 ± 0.05. The proton irradiation data were described best by a one component fit with only one washout component (T_1/2_ of approximately 30 min) with fraction of 0.73 ± 0.06. They discussed that the derived washout fraction obtained from the two comparative studies (0.50 ± 0.05) was significantly lower than 0.73 ± 0.06 by more than two standard deviations. It was concluded that in their offline PET study, there was a larger undetected faster fraction (i.e. fraction = $\:{1-M}_{m}-{M}_{s}$) in the ^12^C ion irradiation data more than in the proton irradiation data. These results showed that the irradiation-induced *β⁺*-emitting nuclides due to autoactivation of the beam experienced washout more rapidly than activation products of the tissue did. This implies that differences between ^12^C ion and proton beams have to be considered for biological washout modeling. Although the specific hot chemistry of the isotopes formed in a living body is still unknown, experimental study by Ammar et al. [36] provides a starting point for further investigation into the dependence of washout parameters on projectile type.

Based on these findings, Bauer et al. [37] investigated a patient data-driven refinement of the biological washout model parameters. In their study, only the slow washout component of the ^11^C contribution was considered, as patient scans are acquired using offline PET measurements. The biological washout parameters $\:{M}_{s}$ and $\:{\tau\:}_{s}$, were determined by fitting the activity decay observed in dynamically reconstructed measured PET data following proton treatment, across three brain tissue categories: white matter (WM), grey matter (GM) and cerebrospinal fluid (CSF). The decay of the mean activity averaged over all voxels assigned to each tissue category, was described by:

13 $$ \left\langle {A_{i} } \right\rangle \left( t \right) = \left\langle {A_{{0,i}}^{{11C}} } \right\rangle \cdot M_{{s,i}} \cdot \exp \left( { - \left( {\lambda _{{11C}} + \lambda _{{s,i}} } \right) \cdot t} \right) $$

where $\:{A}_{0,i}^{11C}$ represents the initial activity contribution from ^11^C decay at the start of the PET measurement as obtained from the MC simulation. $\:{M}_{s,i}$ and $\:{\lambda\:}_{s,i}$ to be determined in the tissue categories *i* = {GM, WM, CSF, the sum of GM, WM and CSF assigned voxels}. Their refined model yields a generally higher similarity for most of the patients, except in highly pathological areas leading to tissue misclassification. They concluded that using washout model parameters deduced from clinical patient data could considerably improve the activity profile similarity for all patients.

## Utilization of the washout effect

### Insights from clinical observations

Biological washout effect is considered an undesirable effect that disrupts the correlation between obtained PET activity distribution and planned dose distribution for PET-based range verification; however, it can provide valuable insights into tumor pathophysiological changes in response to irradiation. Clinical studies have reported apparent changes in biological washout rates in irradiated regions during fractionated irradiation of carbon and proton therapy [13, 14].

Fiedler et al. [13] investigated the tissue-dependent biological washout effect by analyzing data of 50 patients that were obtained at GSI. They reported a reduction in the effective half-life of the irradiation-induced *β*^*+*^-emitting nuclides over the period for the fractionated irradiation of the ^12^C ion therapy. That reduction was rather pronounced in high-dose regions, as illustrated in Fig. 4. They concluded that the observed decrease in biological T_1/2_ was most likely due to increased tumor perfusion over the treatment time. This phenomenon may be related to tumor reoxygenation during radiation therapy [13], such as the evinced by the reopening of temporarily closed blood vessels, improved microcirculation due to reduced tumor pressure, or a decrease in inter-capillary-distance resulting from tumor-volume shrinkage induced by irradiation. Fiedler et al. [13] is the first to report the influence of overall treatment time on the biological washout effect, indicating a pathophysiological response of tissue to irradiation. Fiedler et al.’s study, however, was limited to patients with chordoma or chondrosarcoma of the skull base, which are characterized as slow-growing and late-responding tumors. If their clinical study were extended to tumors with faster dynamics, a more significant trend might have been expected.

Nishio et al. [14] used proton therapy in a clinical study that carried out in-beam PET acquisition in 48 patients with H&N, liver, lung, prostate, and brain cancers. They reported one patient with liver cancer showed a change in the biological washout rate during the treatment period. At the beginning of treatment, the biological washout rate in the necrotic tumor area, identified through histopathological examination, was slower than that in the non-necrotic tumor area. Then, the biological washout rate gradually increased during fractionated proton dose delivery. They interpreted this result as coming from the increase in the biological washout rate in the necrotic area that was caused by a reduction in the number of necrotic cells due to the increased blood flow induced by repeated dose irradiation. These clinical findings [13, 14] highlight the importance of tailoring treatment strategies to individual tumor responses, as reflected by dynamic changes in the biological washout effect.

**Fig. 4:**
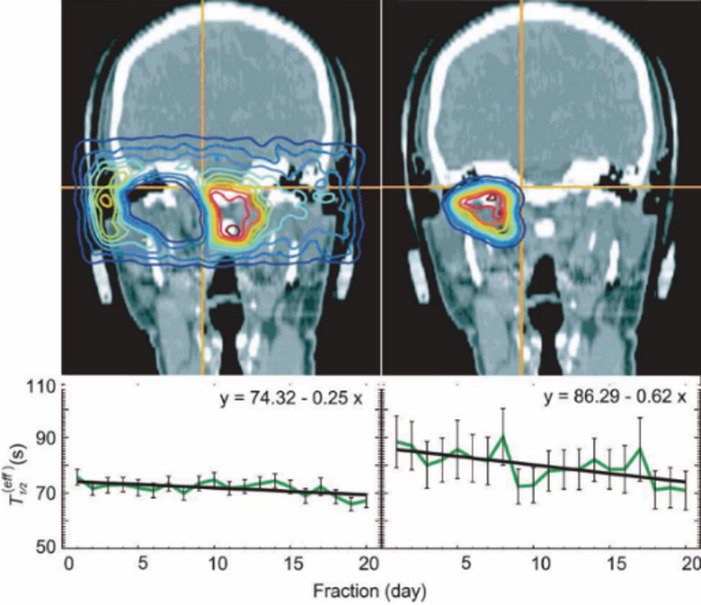
Effect of treatment time on effective half-life by dose: 0–90% (left), 90–100% (right). Figure is reprinted from the work of Fiedler et al., © The Author 2008 licensed under CC BY 4.0 [13]

### Animal studies exploring potential biomarkers

#### Dynamic map of ^15^O production

Feasibility studies of small animals have been conducted to investigate the utilization of biological washout effect as an indicator of tumor response to irradiation. Grogg et al. [17] assumed that the biological decay curves observed immediately after proton irradiation mainly reflected the washout of ^15^O-labeled H_2_O, with minor contributions from longer-lived radionuclides contaminants as ^15^O ions are the dominant source of the PET signal. Because ^15^O ions are produced exclusively through tissue target fragmentation (soft tissue contains of approximately 65–75% water, i.e. oxygen), this approach opened the possibility for developing a dynamic model capable of mapping ^15^O production and tracking changes in biological washout rates throughout treatment. This concept was successfully demonstrated in proton irradiation to rabbit thigh with offline PET acquisitions. Given the predominance of ^16^O in tumor tissue and its critical role in radiation response (e.g., in relation to tumor hypoxia) [38], monitoring of ^15^O ions may provide valuable pathological insights, including information on vascular perfusion and oxygenation within tumor tissues.

#### Detection of tumor vascular status

Following the study by Grogg et al. [17], Toramatsu et al. [19] conducted an in-beam PET study with ^15^O ion beam irradiation of a tumor rat model. Two types of tumor vascular conditions, viable and hypoxic, were modeled by C6 glioma cancer cells implantation into the legs of nude rats. Then the derived medium washout constant, *k*_*2m*_, was compared between normal and tumor tissue across two types of models (Fig. 5). Toramatsu et al. showed that the washout rate in the viable tumor tissue was higher than that in normal tissue, due to tumor angiogenesis leading to vascular hyper-perfusion. In contrast, the washout rate in the hypoxic tumor tissue was lower than that in normal tissue, which was attributed to the lack of vascular structure. Toramatsu et al.’s study demonstrated the feasibility of biological washout-based tumor diagnosis by discriminating against hypoxic tumors from viable tumors in a rat model using washout rate as a new biomarker for tumor vascular status.

Tumor response to radiation is influenced not only by the phenotype of tumor cells but also by the tumor vascular condition, particularly in terms of blood perfusion and hypoxia [39, 40]. In clinical cancer therapy, dynamic contrast-enhanced MRI (DCE-MRI) has been frequently used to assess tumor vasculature perfusion and permeability [39–42]. This technique involves the administration of a gadolinium (Gd)-based contrast agent, followed by the acquisition of MR images with high temporal resolution. In DCE-MRI, a kinetic compartment model is applied to the resulting time-intensity curves (TICs) to obtain quantitative information on tumor vascular perfusion and permeability. Toramatsu et al. [18] conducted a comparative study of washout rates between in-beam PET and DCE-MRI in a tumor rat model. Their study revealed a linear correlation between *k*_*2m*_ values obtained by an in-beam PET experiment followed by ^12^C ion irradiation and those obtained by DCE-MRI followed by use of a Gd contrast agent, in tumor rat models with various vascular conditions. This correlation suggested that the biological washout parameters derived from the irradiation-induced *β*^*+*^-emitting nuclides may also be useful to assess tumor vasculature perfusion and permeability.

**Fig. 5:**
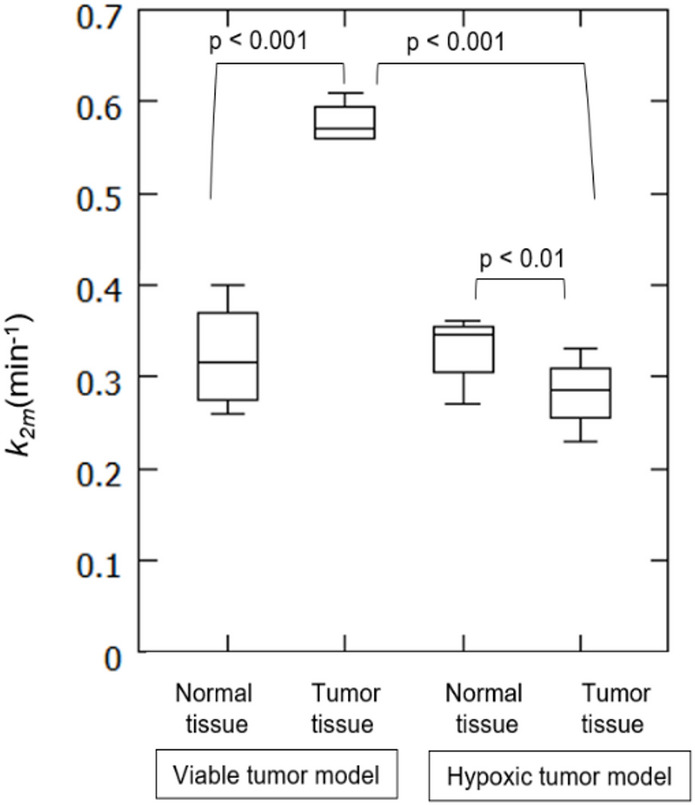
Comparison of washout rate coefficient *k*_*2m*_, the middle washout component, between normal and tumor tissues in irradiated rats from a viable tumor model and a hypoxic tumor model

As described in Sect. 2, the RI beam is useful to track the *β⁺*-emitting nuclides in a living body and it has been used to investigate the mechanism of the biological washout effect. Recently, the Biomedical Applications of Radioactive Ion Beams (BARB) project [43] was launched at GSI/FAIR (Facility for Antiproton and Ion Research) with the aim of performing cancer treatments with RI beams. RI beams have the same biological effects as their corresponding stable ions beams [44, 45], while significantly improving the PET signal-to-noise ratio and reducing the spatial discrepancy between the activity and dose peaks [10]. Within the BARB project, Boscolo et al. [20] successfully treated mice with osteosarcoma using an ^11^C ion beam. They exposed the mice tumors with dose of 5–20 Gy and performed in-beam PET imaging. They applied the multiple components model for the washout effect (faster and slower components) correction, with the following equation to fit the activity data:

14 $$ \begin{gathered} A_{{eff}} \left( t \right) = A_{0} \sum {\:_{i} } w_{i} \exp \left( {\frac{{ - \ln 2}}{{T_{{1/2i}} }}\:t} \right) \hfill \\ \quad {\kern 1pt} \quad \quad \quad \left[ {W_{s} \exp \left( { - k_{s} \:t} \right) + (1 - W_{s} )\exp \left( { - k_{f} \:t} \right)} \right] \hfill \\ \end{gathered} $$

where A_0_ is the activity at the end of the irradiation, $\:{W}_{s}$ is the relative weight of the slower component, and $\:{k}_{f}$ and $\:{k}_{s}$ are the faster and slower washout rate constants, respectively. $\:{T}_{1/2i}$ represents the physical T_1/2_ of the *i*-th contributing radioisotope, such as ^10^C, ^11^O, and ^15^O, and $\:{w}_{i}$ denotes its fractional contribution. In Boscolo *et al.’*s study, a significant difference was observed between the low-dose (5 Gy) and high-dose (20 Gy) irradiation experiments (Fig. 6). The faster component, which was clearly present at low-dose irradiation, essentially disappeared at high-dose irradiation. In another paper by Boscolo et al. [20] they discussed how rapid vascular damage induced by high-dose radiation may delay the biological washout rate. As the tumor damage at high doses induces vascular injury, potentially mediated by an ischemic–reperfusion mechanism [46], Boscolo et al. concluded that their results were consistent with an ischemic stress occurring very early after high-dose exposure [20]. Although reperfusion was not observed within the obtained time interval, the possibility could not be ruled out that it might occur later. Their study demonstrated that the biological washout effect provides a direct evaluation of the vascular perfusion in tumors and thus can help clarify the vascular engagement following radiation therapy.

**Fig. 6:**
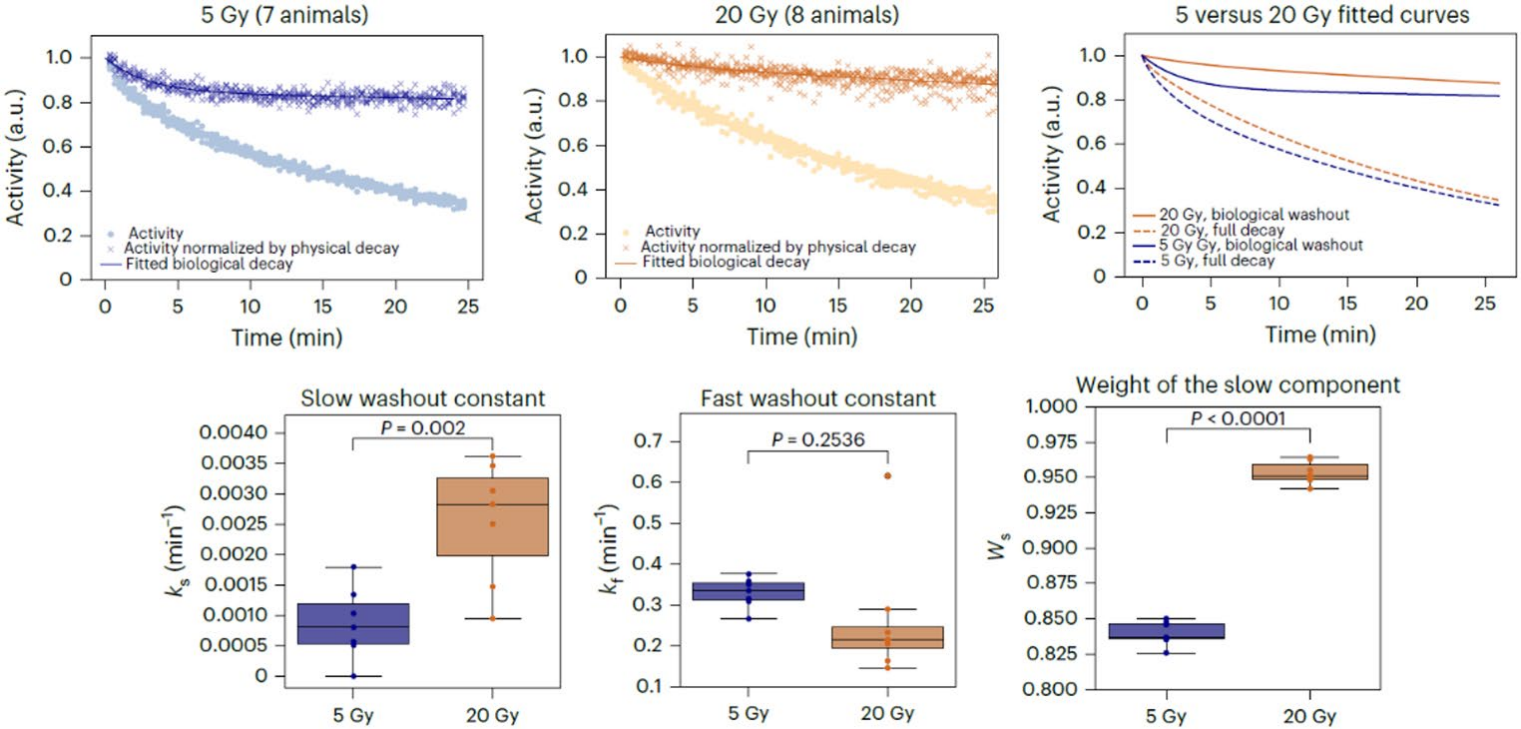
Biological washout rate dependence on irradiated dose. Top: Grouped post-irradiation activity data (left, 5 Gy; middle, 20 Gy; right, fit comparison). Filled circles show total activity; crosses show activity normalized to physical decay. Bottom: Box plots of slower (*k*_*s*_) and faster (*k*_*f*_) washout rates, and slower component weight (W_s_) for 5 Gy (blue) and 20 Gy (orange). Figure is reprinted from the work of Boscolo et al., © The Author 2025 licensed under CC BY 4.0 [20]

## Conclusion and perspectives

Irradiation-induced *β*^*+*^-emitting nuclides offers a promising surrogate signal for verifying the delivered dose in charged particle therapy. However, accurate comparison of an obtained PET activity distribution with the corresponding dose distributions in real clinical scenarios remains challenging due to the significant biological washout effect. Understanding the mechanism of the biological washout effect and improving its modeling are essential, and there is rapid progress in them nowadays. Despite being undesirable in PET-based range verification, the biological washout effect can also provide valuable insights into tumor pathophysiology. Several studies suggested that biological washout parameters could serve as indicators of vascular status within the tumor tissue. A new potential application of PET-based range verification is its ability to monitor changes of tumor vascular status without using any PET tracers. Incorporating biological washout analysis into routine radiation therapy may provide beneficial information for treatment assessment and early prediction of outcomes.

## Data Availability

The data that support the findings of this review are openly available in references.
